# Nutritional prospects of jackfruit and its potential for improving dietary diversity in Uganda

**DOI:** 10.1186/s13104-022-05916-5

**Published:** 2022-02-22

**Authors:** Judith S. Nantongo, Juventine B. Odoi, Hillary Agaba, Samson Gwali

**Affiliations:** National Forestry Resources Research Institute, Kifu, Mukono, Uganda

**Keywords:** Jackfruit, Intraspecific variation, Malnutrition, Plant breeding

## Abstract

**Objective:**

A sustainable way of providing essential nutrients from crops for the poor and undernourished is biofortification, through plant breeding. This study characterised the intraspecific variation of selected nutritional elements in the flakes and seeds of Ugandan jackfruit (*Artocarpus heterophyllus*) plus the phytochemical composition of leaves. The overall aim was to explore possibilities of selecting for varieties that are higher in selected essential nutrients. Selecting for nutrient dense crops has been mostly done for annual agricultural crops, and rarely for perennial fruit trees.

**Results:**

Uganda’s Jackfruit has high macronutrients, especially magnesium and calcium. This study revealed that the amounts of these macronutrients were higher than those found in commonly consumed fruits, giving jackfruit a nutritional advantage with respect to these nutrients. The varieties sampled also differed significantly (p < 0.01) for some nutrients such as vitamin C, crude fat, crude fibre, total soluble solids and juice yield, highlighting the potential for selection for targeted nutritional gains. The seeds however, had less amounts of most of the quantified nutrients that also differed among the varieties. Significant intraspecific variation of the leaf total phenolics was also observed. With regard to the quantified nutritional elements in the flakes, the ethno-varieties were separated in space along PC1 (p < 0.001), PC2 (p < 0.001) and PC3 (p < 0.01) indicating their distinctness.

**Supplementary Information:**

The online version contains supplementary material available at 10.1186/s13104-022-05916-5.

## Introduction

Dietary diversification, supplementation, fortification and biofortification of crop plants are the approaches that are usually proposed for alleviating malnutrition [1, 2], a malaise which occurs in approximately 2 billion people globally and afflicts up to 16% of households in Uganda [3, 4]. Biofortification, the enhancement of nutrient concentration in crops through conventional and modern breeding approaches, agronomic practices as well as genetic engineering [1, 2], is one of the most sustainable and cost-effective approaches to alleviate micronutrient malnutrition globally [2]. Breeding approaches can be an efficient approach provided sufficient genetic variation exists in the crops containing the nutrients of interest. Through crop breeding, significant success has been achieved, for example, in biofortification of lysine, maize and sweet potato [2]. The crops with increased concentrations of essential nutrients, if deployed to consumers through traditional agricultural practices provides a sustainable way of reaching undernourished and low income group families with limited access to diverse diets, supplements, and other fortified foods [2]. It has been suggested that some neglected and underutilized crops (NUCs) that are found in traditional home gardens are nutrient rich and thus potential contributors to the improvement of food security and nutrition [5]. For promising NUCs, understanding intraspecific diversity can guide the selection and promotion of genotypes that meet specific nutritional requirements.

Jackfruit (*Artocarpus heterophyllus* Lam.) has potential to reduce food and nutrition insecurity for both rural and urban communities in various parts of the world [6, 7]. Studies have shown that jackfruit is rich in both macro and micronutrients including carbohydrates, proteins, vitamins as well as minerals that could be a valuable part of food [6, 8]. Both the seeds and the fleshy perianth tissue (often referred to as “flakes”) of jackfruit are consumed as curries and boiled forms. Other parts of jackfruit trees including fruits, leaves, and bark have also been extensively used in traditional medicine due to their anti-carcinogenic, antimicrobial, antifungal, anti-inflammatory, wound healing, and hypoglycemic effects [8–10]. However, the concentration of the chemical compounds potentially varies among jackfruit varieties [8], which are commonly classified according to texture into hard and soft types [8] or colour [11, 12].

In Uganda, jackfruit is still an underutilised and neglected fruit tree species (http://africanorphancrops.org/databases/) and a wide gap exists between production and the marketing of jackfruits for additional income as well as food and nutritional security. This could be related to the lack of knowledge of the nutritional values of the fruit as well as the intraspecific differences that may constrain access to appropriate varieties. Therefore, the objectives of the study were to; (1) characterise the nutritional and phytochemical composition of jackfruit; (2) examine intraspecific differences in the nutritional and phytochemical composition of traditionally identified jackfruit ethno-varieties.

## Main text

### Sampling

**Fig. 1:**
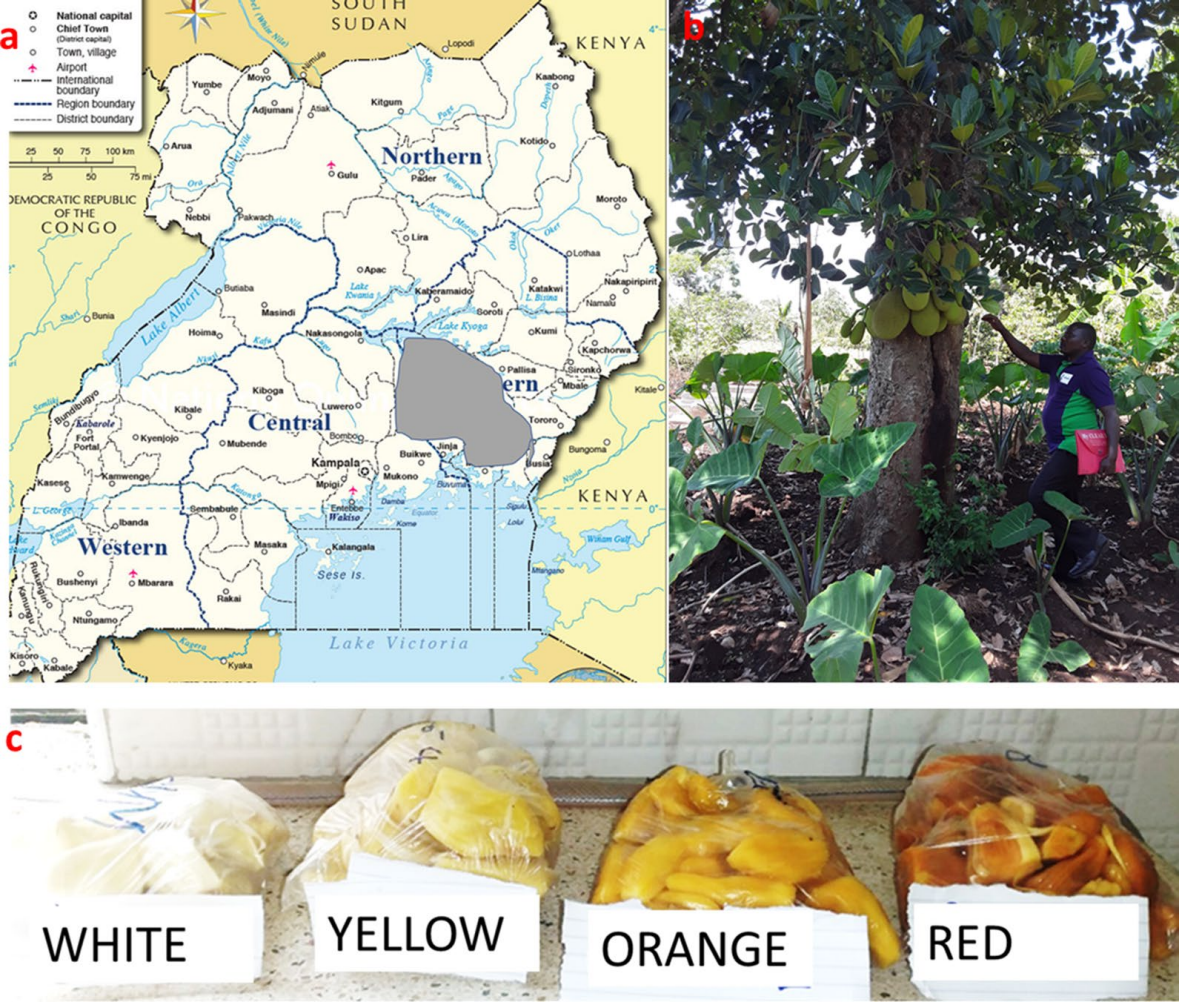
**a** The map of Uganda (https://www.nationsonline.org/oneworld/map/uganda-administrative-map.htm) showing the districts (shaded grey) where jackfruits were sampled, **b** a jack fruit tree in the field and **c** the four colour variants used to define the ethno-varieties of jackfruit in Uganda. The other variant (soft) not shown in the figure is based on texture

Jackfruits were obtained from three districts located in the central and eastern regions of Uganda (Fig. 1) where the trees are mostly grown [11]. The tree species was identified by National Forestry Resources Research Institute (NaFORRI) research team. Guided by farmers, a minimum of 9 mature fruits from each ethno-variety, that is, soft, firm yellow, firm red and firm white [11, 12] as well as another colour variant i.e. orange were collected (Fig. 1). The different ethno-varieties were established in a gene bank at NaFORRI for future studies. The flakes were extracted for each ethno-variety (henceforth referred to as “variety”) in the lab and the fruits further reclassified visually by colour matching.

### Nutritional and phytochemical analysis

The flake and seed samples were analysed for proximate composition (crude proteins, crude fat, crude fibre, total reducing sugars), minerals (calcium, magnesium) and vitamins (vitamin A and C), pH, juice yield, and total titratable acidity (Additional file 1: Methods S1, S2). The leaf samples were analysed for total phenolics and total alkaloid content. All amounts of the different elements were determined using standard analytical procedures according to the Association of Official Agricultural Chemists—AOAC [13] (Additional file 1: Table S1). The flake and leaf samples were analysed on fresh basis to avoid thermal degradation of biochemicals. However, the seeds were allowed to dry for the removal of the seed coat. All analyses were conducted at room temperature.

### Data analysis

The mean values and the associated standard error as well as the Pearson’s phenotypic correlations between all assayed nutritional and mineral elements were estimated using Microsoft Excel. To detect the differences among the different varieties, analysis of variance (ANOVA) for each element was done in R computing version 3.6.0 [14]. Where a significant difference between groups was detected, the least significant difference between the group means at p < 0.05 was calculated, with Bonferroni adjustment for multiple testing. To test whether the varieties could be distinguished using multiple nutritional elements, a multivariate principal component analysis (PCA) was done in R using FactoMineR function [15]. To identify the significance of the separation along the principal components, ANOVA was applied to the loadings of the first three principal components. Differences between groups were tested as mentioned above.

### Results and discussion

#### Nutritional elements in jackfruit flakes

On average, most nutritional elements of jackfruit assessed in this study were comparable to those of commonly consumed fruits such as banana, mangoes or pineapple [16], except for amount of vitamin C that was lower than for most of the other fruits (Table 1). However, jackfruit had higher calcium (Ca) and magnesium (Mg) contents, suggesting that it could be an important source of these mineral elements. Ca and Mg are macro elements that are necessary for proper development of bone and structural tissue formation and play important roles in glucose and protein absorption and metabolism [17]. They are also involved in the regulation and dilation of blood vessels and a regular heartbeat [17]. Deficiency of Ca and Mg may cause weak bones and structural connective tissue formation, hypertension, and poor glucose absorption and utilization. Therefore, exploiting the comparative nutritional advantages of jackfruit can boost consumption, food security and nutritional outcomes of households for these particular minerals. It is noted that although jackfruit may be an important source of these nutrients, the rest of the fruits, continue to be the major sources of other important nutrients. Therefore, diversification of fruit consumption is vital for optimal acquisition of essential minerals and vitamins. This points to the importance of on-farm fruit diversity as a key strategy to alleviate malnutrition in small-holder farmers [18, 19].

**Table 1:** Average nutritional elements of jackfruit flakes and selected fruits

	Magnesium mg/100 g	Calcium mg/100 g	Vitamin A µg/100 g	Vitamin C mg/100 g	Sugars mg/g	Proteins g/100 g	Fats g/100 g	Fibre g/100 g
Jack fruit flakes*	83	60	10	9	29	0.7	0.4	1.4
Jack fruit seeds*	95	45	na	4.3	na	0.4	0.2	2.3
Avocado	29	13	7	10	0.7	2	14.7	6.7
Bananas	27	5	3	8.7	12.2	1.1	0.3	2.6
Oranges	10	40	11	53.2	9.4	0.9	0.1	2.4
mangoes	10	11	54	36.4	13.7	0.8	0.4	1.6
pineapple	12	13	3	16.9	9.9	0.6	0.1	1.4
Watermelon	10	7	28	8.1	6.2	0.6	0.2	0.4
Guavas	22	18	31	228.3	8.9	2.6	1	5.4
Papaya	21	20	47	60.9	7.8	0.5	0.3	1.7
Passion fruit	29	12	64	30	12.1	2.2	0.7	10.4
Tamarind	92	74	2	3.5	38.8	2.8	0.6	5.1
Jackfruit	29	24	5	13.7	19.1	1.7	0.6	1.5

The amount with asterisk was estimated in this study as an average of triplicate samples while the other amounts were derived from USDA National Nutrient Data Base [16]

*Na* not assessed

The proximate composition of jackfruit flakes was in range or lower than what has been established for other fruits, except for the higher amounts of sugars [16] (Table 1). The relative nutritional composition for jackfruit in this study differed from other jackfruit estimates. For example, in Nigeria, jackfruit in Nigeria has been characterised with very high values of Vitamin A in the flakes (294.8 µg/100 g) as well as high Vitamin C content (27.6 mg/100 g). In contrast, the calcium and magnesium contents of the pulp were low, estimated at 19.3 and 29.6 mg/100 g [20]. Although the method of assessment may be important in the comparative ability of these values [21], the results could also suggest that differences in the environment affect nutritional composition of jackfruits.

#### Correlations between nutritional elements of jackfruit flakes

To elucidate whether jackfruit can simultaneously provide more than one nutritional element, most of the Pearson’s phenotypic correlations among the nutritional elements were low (Additional file 2: Table S2), except for the perfect correlation between crude fibre and crude fat (r^2^ = 1.00). A higher juice yield may also be associated with a lower pH (r^2^ = − 0.64). Overall, based on the assayed traits, jackfruit may not be a source of multiple elements. Correlations are also particularly important when selecting germplasm for improvement. Positively correlated nutrients can be simultaneously improved, hence the results here may suggest that multiple trait selection may not be feasible for the assayed traits, except for the amounts of fibre and fat. Nevertheless, characterising genetic correlations can be more informative than the phenotypic correlations that have been presented in this study [22].

#### Intraspecific variation of nutritional elements of jackfruit flakes

Some of the nutritional elements in the flakes varied significantly among the different varieties (Additional file 3), for example, vitamin C was higher in the ‘orange’ variety while the ‘soft’ variety, had a higher juice content compared to other varieties. Similarly, the ‘yellow’ variety had a higher ash content than the others, suggesting that this variety may on a whole have a higher level of inorganics, macro and essential elements, and other minerals [23]. Carotenoids are known to impart yellowish-red colour to many foods and their ratio is supposed to render the fruits the various yellow to orange shades of colour [24]. Therefore, characterizing the amount of carotenoids as potential distinguishing elements between the different varieties is encouraged. Understanding the intraspecific variation of the nutritional elements among the varieties highlights the opportunity to select particular varieties as sources of specific nutritional elements. It also points to the potential for developing tailored value-added products. The high and low amounts of nutritional elements were not consistent among the varieties, for example the ‘white’ variety that tended to have the highest magnesium had the lowest calcium content. This suggests that optimal nutritional benefit from jackfruit will be derived by consuming products from diverse varieties. However, breeding efforts to harness the different nutrients into a single variety, aspects of biofortification, are also possible [25, 26].

**Fig. 2:**
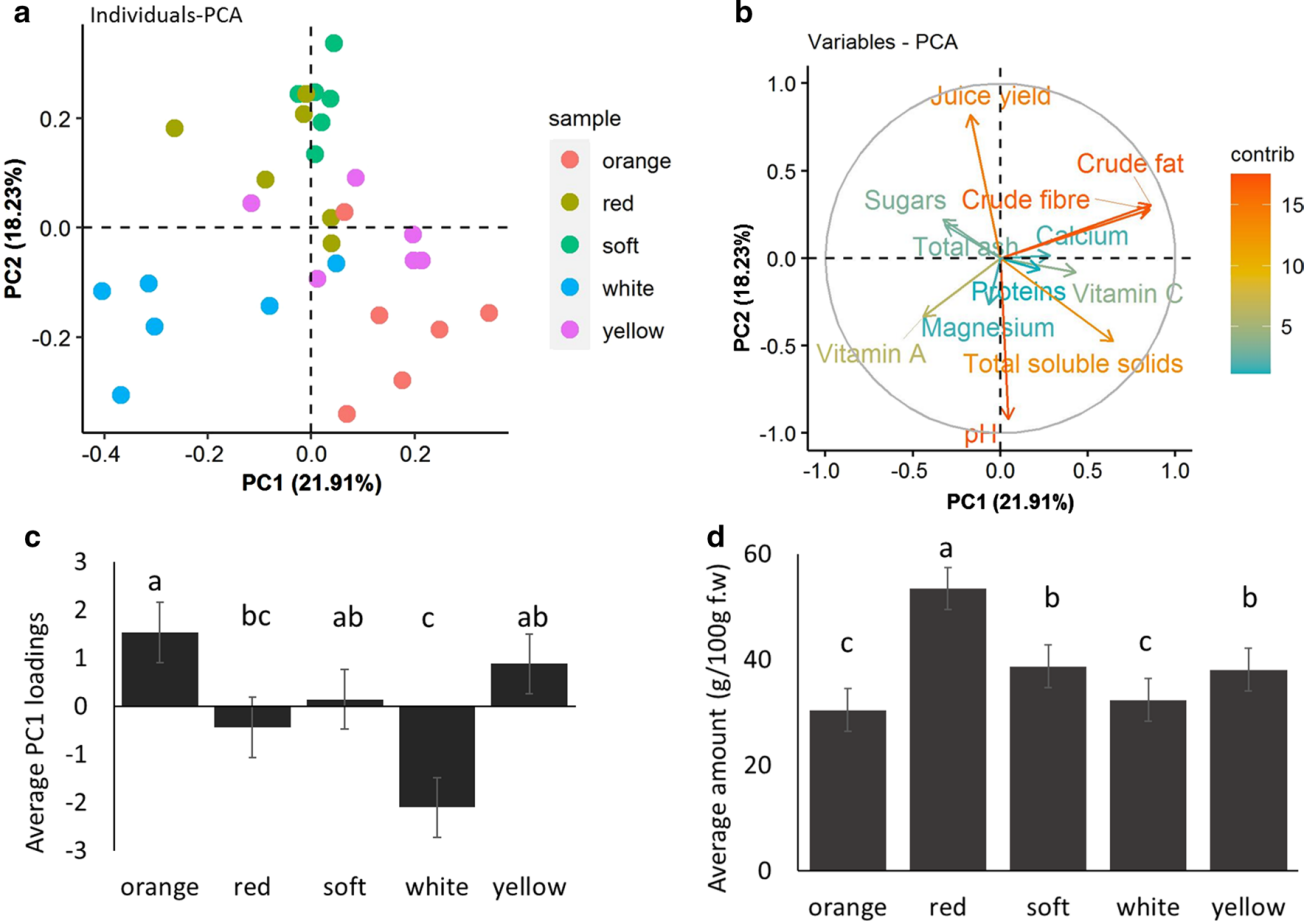
**a** Plot of the first (PC1) and second (PC2) principal component for jackfruit samples based on the nutritional composition of the flakes. PC1 explained 21.91% and PC2 explained 18.23% of the variation, **b** the biplot showing calcium and magnesium as the strongest predictors of the variation at PC1 and PC2, **c** Average loadings of PC3 differentiating the orange and white varieties. Different letters adjacent to the plot indicate significant differences (p < 0.05) between the varieties based on the least significant difference with Bonferroni adjustments. Average loadings of PC1 differentiating the orange and white varieties. Different letters adjacent to the plot indicate significant differences (p < 0.05) between the varieties based on the least significant difference with Bonferroni adjustments, **d** Average amount of total phenolics in the leaves of the different jackfruit varieties. Different letters adjacent to the plot indicate significant differences (p < 0.05) between the varieties based on the least significant difference with Bonferroni adjustments

To test whether the varieties could be separated in space based on the suite of chemical compounds, a principal component analysis (PCA) plot (Fig. 2a) showed the varieties were clearly separated along principal components (PC), PC1 (p < 0.001) and PC2 (p < 0.001). The yellow’ and ‘orange’ varieties were also distinguishable along PC3 (p < 0.01). The biplot (Fig. 2b) indicated juice yield, pH, crude fat and fibre as the strongest predictors of the varieties.

#### Nutritional elements in jackfruit seeds

The nutritional value of seeds differed from those of the flakes (Table 1). For example, two-fold vitamin C, proteins and crude fat content were found in the flakes than the seeds. This contrasts other studies that have shown more amounts of most nutritional elements in seeds than flakes [20]. On the contrary, more magnesium and fibre were found in the seeds than in the flakes, suggesting that seeds can be a better nutritional source of these elements compared to flakes and could be incorporated in food products. Similarly, the nutrients varied among the varieties, for example the amount of magnesium varied from 84.9 mg/100 g in red to 104.9 mg/100 g in white varieties (Additional file 3), suggesting the potential for enhancement of magnesium in seeds through selection.

#### Phytochemical composition of leaves

The total phenolics were significantly higher in the ‘red’ varieties compared to ‘white’ and ‘orange’ (Fig. 2d). No differences among varieties were detected for alkaloids. A multivariate analysis including flake and leaf chemistry still showed that the varieties were differentiated along PC1 and PC2. The reduction in the contribution of the principal components to the overall variation (Additional file 4: Fig. S1a) suggests that leaf chemistry is also an important source of variation among the varieties. The biplot also identified that total leaf phenolics was an important explanatory factor (Additional file 4: Fig. S1b). Therefore, analysing the phenolics of the flakes may be an important distinguishing feature of the different varieties.

In summary, the study has shown that; (1) jackfruit could be important as a source of selected mineral elements such as magnesium and calcium; (2) the varieties vary in the amounts of the different nutritional and mineral elements; and (3) intraspecific variation of the nutritional elements can enable selection, biofortification and promotion of varieties for targeted nutritional elements.

## Limitations

The variation in the degree of ripening of the analysed samples may have caused colour mislabelling and differences in nutritional composition. Future studies, therefore, need to monitor the fruits so that they are harvested at precise and well-defined physiological states. Increasing sample size as well as improving accuracy in separating the colours of the flakes for example using standard methods like colour charts may give clearer results. Further examination of factors that make jackfruit less palatable such as anti-nutritional factors and high perishability is also highly recommended. A chemical fingerprint performed by for instance chromatographic techniques is highly recommended, for ease of comparability of results across different studies. Determining the extent of genetic control of the amounts of nutritional elements will give an indication of potential genetic gains.

## Supplementary Information

**Additional file 1. ** Additional methods including summary methods used in proximate and mineral analysis of jackfruit.

**Additional file 2.** Correlation between selected nutrition elements in the flakes of jackfruit.

**Additional file 3.** Average amounts (mean ± standard error) of selected nutritional elements in the flakes and seeds of jackfruit. f.w and d.w indicate that the amounts are presented on fresh and dry weight basis respectively. For the amounts quantified in the triplicates of flake and leaf samples, different letters adjacent to the mean indicate significant differences (p < 0.05) between the varieties based on the least significant difference with Bonferroni adjustments. Some mean amounts that were not different have no numbers. For the amounts detected in the seeds, not statistical tests were done since the analysis was done on duplicated samples.

**Additional file 4.** 1(a) Plot of the first (PC1) and second (PC2) principal component for jackfruit samples based on the composition of the flakes and the leaves. PC1 explained 20.38% and PC2 explained 18.47% of the variation, (b) the biplot showing the strongest predictors of the variation at PC1 and PC2.

## Data Availability

The datasets used and/or analysed during the current study available from the corresponding author on reasonable request.

## Abbreviations

NUCs: Neglected and underutilized crops
NaFORRI: National Forestry Resources Research Institute
ANOVA: Analysis of variance
PCA: Principal component analysis
PC: Principal component
