# Supplementary material for: Nutritional prospects of jackfruit and its potential for improving dietary diversity in Uganda
Source: BMC Res Notes. 2022 Feb 22;15:74. doi: 10.1186/s13104-022-05916-5 (PMC8862346; doi:10.1186/s13104-022-05916-5)
Supplement: Supplementary file 1 — Additional file 1. Additional methods including summary methods used in proximate and mineral analysis of jackfruit. [file 13104_2022_5916_MOESM1_ESM.docx]

**Methods S1:**

From each selected tree, one mature fruit that was within 3 to 4 days of full ripening as guided by local knowledge was harvested. A minimum of 9 fruits per ethno-variety (henceforth referred to as ‘varieties’) were collected. The fruits were transported to the Food Biosciences Laboratory of the National Agricultural Research Laboratories, Kawanda for extraction of the flakes and seeds. From each fruit, one subsample of at least 1kg of flakes with seeds was prepared. These subsamples were composited to make final samples that were analysed. The final sample of the flakes was composited from a minimum of three subsamples of the same variety collected from different districts. Each variety had three composited flake samples for the lab analysis. Each sample was analysed in duplicate giving a total of 6 observations per variety. From each tree, leaves were also collected for phytochemical analysis and the lab samples were composited as above to make three composite leaf samples per variety. However, all the seeds from the subsamples were composited to make 1 sample that was analysed in duplicate. All samples were immediately kept at 4^0^C until the chemical extractions and analysis.

**Methods S2:**

**Table S1**: Summary methods used in proximate and mineral analysis of jackfruit

|  | Part analysed | Method | Reference |
| --- | --- | --- | --- |
| Crude protein | flakes, seed | Kjeldahl’s | Lynch and Barbano [1] |
| Crude fat | flakes, seed | Soxhlet extraction | AOAC Official Method 991.36; Thiex, Anderson [2] |
| Crude fibre | flakes, seed | Gravimetric | AOAC Official Method 978.10; Möller, Janjira [3] |
| Total reducing sugars | flakes, seed | Anthrone | Jayaraman [4] |
| Calcium | flakes, seed | atomic absorption flame spectroscopy | Agri-Laboratory Association of Southern Africa |
| Magnesium | flakes, seed | atomic absorption flame spectroscopy | Agri-Laboratory Association of Southern Africa |
| Vitamin A | flakes, seed | chromatography | AOAC Official Method 992.06 |
| Vitamin C | flakes, seed | 2, 6 – dichlorophenol indophenol visual titration | AOAC Official Method 992.06 |
| pH | flakes | Fischer pH meter | Ranganna [5] |
| Juice yield | flakes | Blending | Ranganna [5] |
| Total ash | flakes, seed | Combustion | AOAC Official Method 942.05 Ranganna [5] |
| Total soluble solids | flakes | Refractometer | Ranganna [5] |
| Titrable acidity | flakes | Titration | Ranganna [5] |
| Total phenolics | leaves | Folin-Ciocalteu assay | Ainsworth and Gillespie [6] |
| Total alkaloids | leaves | based on the reaction with bromocresol green | Shamsa, Monsef [7] |

1. Lynch, J.M. and D.M. Barbano, *Kjeldahl Nitrogen Analysis as a Reference Method for Protein Determination in Dairy Products.* Journal of AOAC INTERNATIONAL, 1999. **82**(6): p. 1389-1398.

2. Thiex, N.J., S. Anderson, and B. Gildemeister, *Crude fat, diethyl ether extraction, in feed, cereal grain, and forage (Randall/Soxtec/submersion method): collaborative study.* J AOAC Int, 2003. **86**(5): p. 888-98.

3. Möller, J., et al., *Gravimetric determination of acid detergent fiber and lignin in feed: interlaboratory study.* Journal of AOAC International, 2009. **92**(1): p. 74-90.

4. Jayaraman, J., *Laboratory manual in biochemistry*. 1981: Wiley Eastern Delhi, India.

5. Ranganna, S., *Manual of analysis of fruit and vegetable products*. 1979: Tata McGraw Hill, New Delhi, India.

6. Ainsworth, E.A. and K.M. Gillespie, *Estimation of total phenolic content and other oxidation substrates in plant tissues using Folin–Ciocalteu reagent.* Nature Protocols, 2007. **2**(4): p. 875-877.

7. Shamsa, F., et al., *Spectrophotometric determination of total alkaloids in Peganum harmala L. using bromocresol green.* Res J Phytochem, 2007. **1**(2): p. 79-82.
