# Supplementary material for: Nutritional prospects of jackfruit and its potential for improving dietary diversity in Uganda
Source: BMC Res Notes. 2022 Feb 22;15:74. doi: 10.1186/s13104-022-05916-5 (PMC8862346; doi:10.1186/s13104-022-05916-5)
Supplement: Supplementary file 2 — Additional file 2. Correlation between selected nutrition elements in the flakes of jackfruit. [file 13104_2022_5916_MOESM2_ESM.docx]

**Supplementary Table 2:** Correlation between selected nutrition elements in the flakes of jackfruit

|  | Vitamin A | Vitamin C | Calcium | Magnesium | Protein | Crude fat | Crude fibre | Total reducing sugars | Total ash | Total soluble solids | Juice yield |
| --- | --- | --- | --- | --- | --- | --- | --- | --- | --- | --- | --- |
| Vitamin C | -0.42 |  |  |  |  |  |  |  |  |  |  |
| Calcium | -0.02 | 0.12 |  |  |  |  |  |  |  |  |  |
| Magnesium | 0.29 | 0.03 | 0.15 |  |  |  |  |  |  |  |  |
| Protein | -0.01 | -0.08 | 0.20 | -0.06 |  |  |  |  |  |  |  |
| Crude fat | -0.24 | 0.11 | 0.18 | -0.02 | 0.15 |  |  |  |  |  |  |
| Crude fibre | -0.18 | 0.11 | 0.20 | 0.00 | 0.15 | 1.00 |  |  |  |  |  |
| Total reducing sugars | -0.15 | 0.03 | -0.26 | -0.13 | -0.22 | -0.16 | -0.20 |  |  |  |  |
| Total ash | -0.04 | -0.07 | 0.09 | -0.18 | -0.11 | -0.21 | -0.21 | 0.27 |  |  |  |
| Total soluble solids | -0.35 | 0.40 | -0.04 | -0.21 | -0.08 | 0.34 | 0.33 | -0.12 | -0.05 |  |  |
| Juice yield | 0.06 | -0.19 | -0.11 | -0.31 | -0.12 | 0.12 | 0.12 | 0.00 | 0.02 | -0.49 |  |
| pH | 0.43 | -0.05 | -0.08 | 0.12 | 0.06 | -0.15 | -0.13 | -0.14 | -0.20 | 0.50 | -0.64 |
