# Supplementary material for: Nutritional prospects of jackfruit and its potential for improving dietary diversity in Uganda
Source: BMC Res Notes. 2022 Feb 22;15:74. doi: 10.1186/s13104-022-05916-5 (PMC8862346; doi:10.1186/s13104-022-05916-5)
Supplement: Supplementary file 3 — Additional file 3. Average amounts (mean ± standard error) of selected nutritional elements in the flakes and seeds of jackfruit. f.w and d.w indicate that the amounts are presented on fresh and dry weight basis respectively. For the amounts quantified in the triplicates of flake and leaf samples, different letters adjacent to the mean indicate significant differences (p < 0.05) between the varieties based on the least significant difference with Bonferroni adjustments. Some mean amounts that were not different have no numbers. For the amounts detected in the seeds, not statistical tests were done since the analysis was done on duplicated samples. [file 13104_2022_5916_MOESM3_ESM.docx]

**Supplementary Table 3:** Average amounts (mean ±standard error) of selected nutritional elements in the flakes and seeds of jackfruit. f.w and d.w indicate that the amounts are presented on fresh and dry weight basis respectively. For the amounts quantified in the triplicates of flake and leaf samples, different letters adjacent to the mean indicate significant differences (p < 0.05) between the varieties based on the least significant difference with Bonferroni adjustments. Some mean amounts that were not different have no numbers. For the amounts detected in the seeds, not statistical tests were done since the analysis was done on duplicated samples.

|  | Orange | Red | Soft | White | Yellow |
| --- | --- | --- | --- | --- | --- |
| **Flakes** |  |  |  |  |  |
| Juice yield (% w/w) | 0.06±0.00^c^ | 0.09±0.01^ab^ | 0.10±0.01^a^ | 0.08±0.00^bc^ | 0.08±0.00^abc^ |
| Vitamin A (µg/100g f.w) | 8.31±0.25 | 8.09±0.92 | 9.32±0.60 | 14.29±3.42 | 10.35±0.68 |
| Vitamin C- (ascorbic acid) (mg/100g f.w) | 12.00±1.06 ^a^ | 10.55±2.16 ^ab^ | 8.07±1.95 ^ab^ | 5.32±0.67 ^b^ | 9.02±0.55 ^ab^ |
| Calcium (mg/100g f.w) | 67.95±2.89 | 58.57±5.04 | 62.72±7.32 | 54.28±13.78 | 58.60±15.42 |
| Magnesium (mg/100g f.w) | 81.02±15.54 | 77.03±4.52 | 86.03±6.17 | 88.73±6.96 | 84.50±4.01 |
| Proteins (g/100g f.w) | 0.44±0.02 | 0.46±0.01 | 0.42±0.01 | 0.40±0.02 | 1.93±1.50 |
| Crude fat (g/100g f.w) | 0.50±0.08 ^a^ | 0.36±0.07 ^ab^ | 0.54±0.04 ^a^ | 0.16±0.05 ^b^ | 0.49±0.07 ^a^ |
| Crude fibre (g/100g d.w) | 1.50±0.08 ^a^ | 1.36±0.07 ^ab^ | 1.54±0.04 ^a^ | 1.18±0.05 ^b^ | 1.49±0.07 ^a^ |
| Total reducing sugars (mg/g d.w) | 290.66±1.21 | 290.78±1.11 | 291.29±1.20 | 291.12±1.72 | 288.98±0.61 |
| Total ash (%) | 0.42±0.02 ^a^ | 0.47±0.01 ^a^ | 0.45±0.01 ^a^ | 0.44±0.01 ^a^ | 0.27±0.02 ^b^ |
| Total soluble solids (% Brix) | 25.9±0.13 ^a^ | 19.77±0.76 ^b^ | 19.33±0.63 ^b^ | 19.77±1.41 ^b^ | 20.7±0.95 ^b^ |
| pH | 5.19±0.06 | 4.57±0.05 | 4.47±0.04 | 5.15±0.05 | 4.87±0.08 |
|  |  |  |  |  |  |
| **Seeds** |  |  |  |  |  |
| Vitamin C-(ascorbic acid) (mg/100g f.w) | 5.10 | 6.05 | 4.40 | 2.55 | 3.50 |
| Calcium (mg/100g f.w) | 48.70 | 40.55 | 39.75 | 64.35 | 31.05 |
| Magnesium (mg/100g f.w) | 84.90 | 92.90 | 104.85 | 108.30 | 86.40 |
| Crude protein (g/100g f.w) | 0.44 | 0.39 | 0.46 | 0.24 | 0.42 |
| Crude fat (g/100g f.w) | 0.23 | 0.12 | 0.21 | 0.29 | 0.38 |
| Crude fibre (g/100g f.w) | 2.32 | 2.29 | 2.31 | 2.32 | 2.16 |
| Total ash content (%) | 0.43 | 0.44 | 0.45 | 0.46 | 0.45 |
|  |  |  |  |  |  |
| **Leaves** |  |  |  |  |  |
| Alkaloids (mg/100g f.w) | 12.09±0.44 | 12.13±0.40 | 11.30±0.46 | 12.87±0.33 | 12.10±0.43 |
| Phenolics (mg/100g f.w) | 30.43±0.87 ^c^ | 53.43±8.60 ^a^ | 38.68±2.23 ^b^ | 32.33±0.95 ^c^ | 38.03±2.19 ^b^ |
|  |  |  |  |  |  |
