# Supplementary material for: Nutritional prospects of jackfruit and its potential for improving dietary diversity in Uganda
Source: BMC Res Notes. 2022 Feb 22;15:74. doi: 10.1186/s13104-022-05916-5 (PMC8862346; doi:10.1186/s13104-022-05916-5)
Supplement: Supplementary file 4 — Additional file 4. 1(a) Plot of the first (PC1) and second (PC2) principal component for jackfruit samples based on the composition of the flakes and the leaves. PC1 explained 20.38% and PC2 explained 18.47% of the variation, (b) the biplot showing the strongest predictors of the variation at PC1 and PC2. [file 13104_2022_5916_MOESM4_ESM.docx]

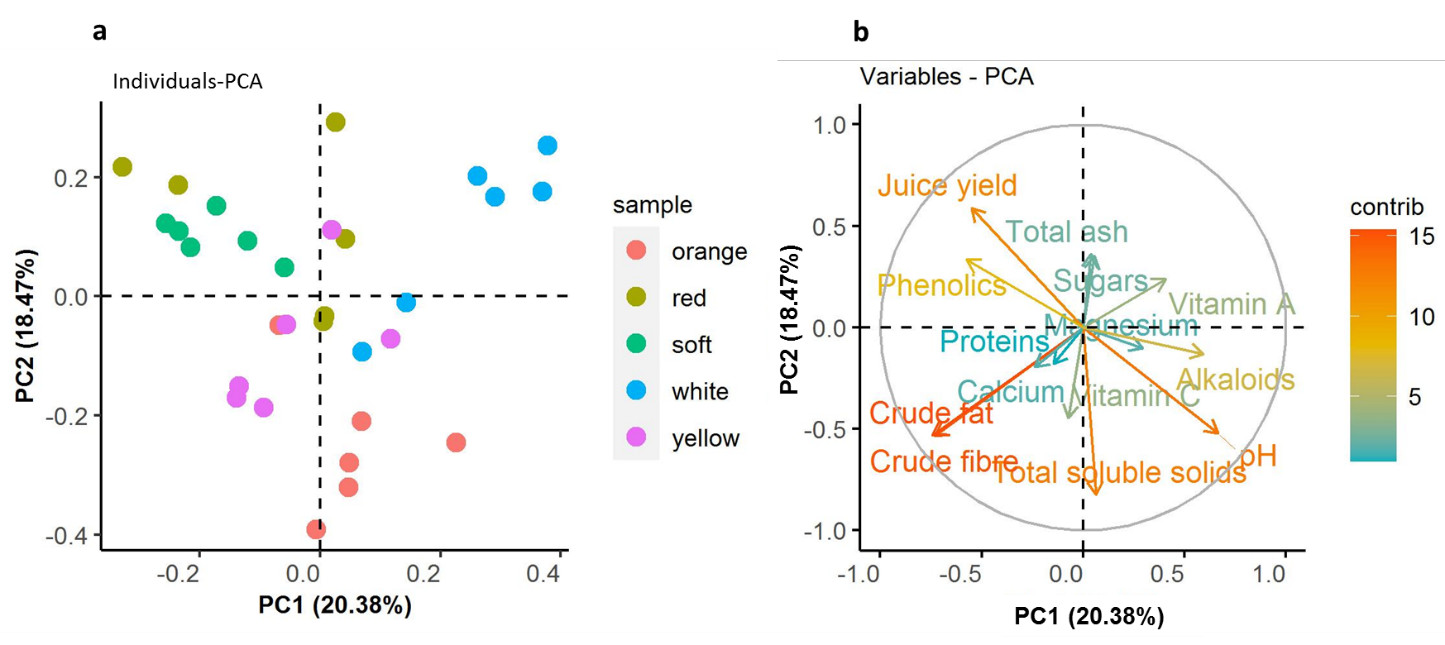


**Supplementary Figure** 1(a) Plot of the first (PC1) and second (PC2) principal component for jackfruit samples based on the composition of the flakes and the leaves. PC1 explained 20.38% and PC2 explained 18.47% of the variation, (b) the biplot showing the strongest predictors of the variation at PC1 and PC2.
